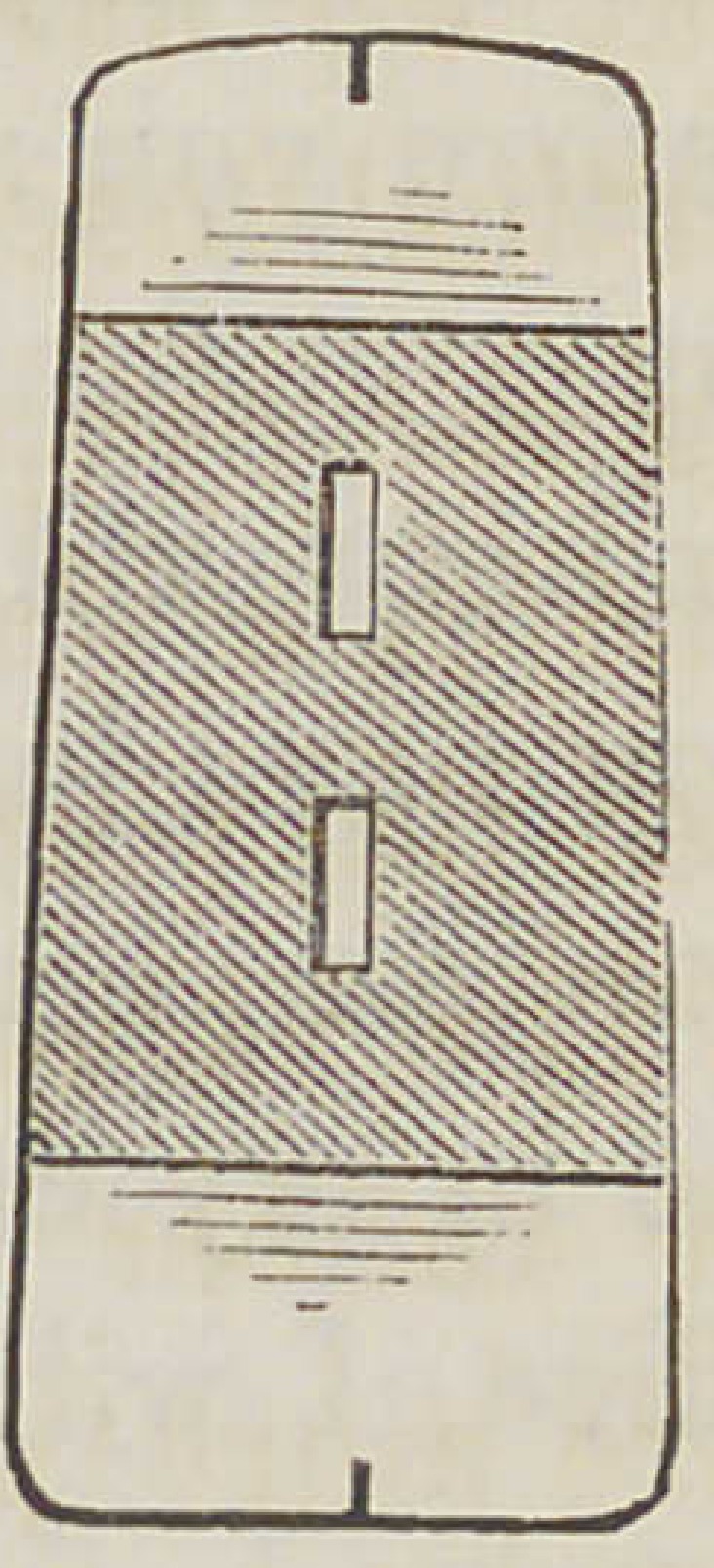# Improvement in Artificial Teeth

**Published:** 1867-07

**Authors:** John C. K. Crooks

**Affiliations:** Birmingham, Michigan


### IMPROVEMENT IN ARTIFICIAL TEETH.

BY JOHN C. K. CROOKS, M. D., BIRMINGHAM, MICHIGAN.

Notwithstanding the great artistic skill attained in the manufacture of artificial teeth, in which the material, form, color, etc., have been brought quite to a state of perfection, still, since the almost universal adoption of vulcanized rubber as a base, there have been several very important things needed, in order to fully meet the requirements of the profession, and to elevate the character of this particular kind of work. In these remarks, I refer more especially to that great need felt by the entire profession of a tooth *embodying a greater amount of strength*—a tooth that will correspond more nearly with the strength of the plate upon which it is inserted. That the teeth manufactured at the present day, for rubber bases, are more frail than those used a few years ago, so generally, upon metallic plates, is universally noticeable, so much so, that no one would now pretend to insert a *single* tooth upon rubber and expect durability, unless he used the old plate teeth, properly backed, etc. Such certainly has been my experience, and such is the advise of those who are known to aim at a high standard in their profession. Now, in what does this difference consist? Our manufacturers are the same, the materials they use have undergone no change, there is undoubtedly all of the old care and pains-taking in their manipulations; where then is the difficulty? I answer, unhesitatingly, *it is in the form and kind of fastening used—the pins being inserted transversely instead of vertically.*

In the use of rubber as a base, it early became a *necessity* to have teeth with the pins arranged transversely, in order to get sufficient strength of rubber and at the same time not expose the pins in finishing, or unduly increase the thickness of the plate. This having been the case, the manufacturer has been compelled, thus far, to furnish teeth for the profession, many of which were nearly *worthless* on account of the almost literal severing of them by this transverse insertion of the pins. A set of plain or single gum teeth may be completed to-day, and to-morrow our patient may return with one or more fractured—not the molars, nor the bicuspids, unless they are very small, but generally the *lateral incisors*, or it may be the *central* or the *canine*, and then *invariably at the point where the pins are inserted into the teeth!* Such was not the case with the old vertical pins; neither is it so now, even in teeth of the smallest sizes. Then, why should this frailty *now* obtain, unless there is something radically wrong somewhere, and *that* the defect already pointed out. That this is the difficulty there cannot be the shadow of a doubt; and if so, what is the remedy? To answer this question to the satisfaction of all, to meet the demands of the profession, our manufacturers of teeth have exhibited commendable zeal, and in trying to do so myself, I cannot but feel the importance of the undertaking and the embarrassment attending it; for I am well aware that however much a remedy may be a pet idea with its originator, or however much he may conceive it to be perfect, yet that great test of value, *actual use*, may prove it to be worthless. Whatever I may have to say, then, in commendation of my device to increase the strength of teeth for rubber bases, is said only with the *hope and belief* that it is a *step* in the right direction.

In experimenting upon this subject, I availed myself, in the begining, of this important hint, that *teeth with vertical pins possess the greatest strength and are better adapted to all purposes for which they are designed than any others.* Bearing this idea prominently in mind, the query arose, how can a "fastening for artificial teeth" be arranged, so that the "teeth portion" may have its greatest diameter correspond to the greatest diameter of the tooth, (longitudinal), while the "rubber portion" may stand horizontally to the plate? With this inquiry, there also arose the consequence of a desire, which might enable the Dentist to replace broken teeth with facility and with permanency.

To meet these two important objects had in view, I hit upon a fastening in the form of an eye—like the eye to a button—said eye being made of platinum wire and provided with a double shank, (the ends of the wire) and so bent that the greatest diameter of the shank is at right angles to the plane of the eye, the eye being as large as the smaller teeth will allow. Then, to still further preserve the material in the transverse diameter of the tooth, and to thus add to its strength, I flattened the ends of the wire constituting the shank, so that its minimum flattening was a little way from the eye, and the maximum at the extremity of the shank, making it fan-shaped, and thereby dovetailing it into the substance of the tooth. With this devise, we can readily see that we have more than met the first object in view — a vertical insertion of the pins into the tooth, and by elongating the eye, making it in form like a staple, we can easily furnish a way to repair broken teeth, by cutting a mortice into the plate and securing the tooth by a vertical pin.

The value of this invention applies to all kinds of teeth for rubber bases, of course, for purposes of repair, but it is sure more particularly in single, plain or gum teeth, where it is of the utmost consequence to have all the strength of the tooth preserved. In the insertion of single teeth, and in those cases where, from the prominence of the jaw, it is necessary to use plain teeth—cutting away the plate and inserting the teeth with the upper extremities applied directly to the gums, the value of the eye attachment cannot be estimated. The fastening into the rubber is immovable, while the increased tooth substance, transversely, greatly enhances its strength—indeed, it may almost be said an hundred fold, when we consider how nearly divided are the smaller teeth by the fastening in use at the present day.

With the above description of my device for the "improvement of artificial teeth," I leave the subject for the consideration of an intelligent and charitable profession. That it meets *every* demand, I do not for a moment pretend. If it contains one idea of value, I am satisfied. Although protected by letters patent, it is free to Dentists *everywhere*, to be used in their own laboratories and for their own purposes.

For a further description, I refer the reader to the accompanying drawings and explanations :

*Fig. 1.*

*Fig. 2.*

*Fig. 3.*

*Fig. 4.*

*Fig. 5.*

*Fig. 6.*

Fig. 1. The ordinary form of the eye as inserted into the tooth by the Manufacturer.

Fig. 2. An eye elongated by the Dentist for the purpose of repairing by inserting into a mortice cut into the plate and retained there by a vertical pin of silver or platina wire.

Fig. 3. An eye with the shank flattened at the extremity to further strengthen the tooth in its transverse diameter and to obtain a dove-tailed hold in the tooth.

Fig. 4. A side view of a tooth showing the vertical position of the double shank in the body of the tooth or eye for insertion in the base.

Fig. 5. A top view of a tooth for the same illustration.

Fig. 6. A vertical section taken in the line X X Fig. 4, showing the vertical position of the shank.

## Figures and Tables

**Fig. 1. f1:**
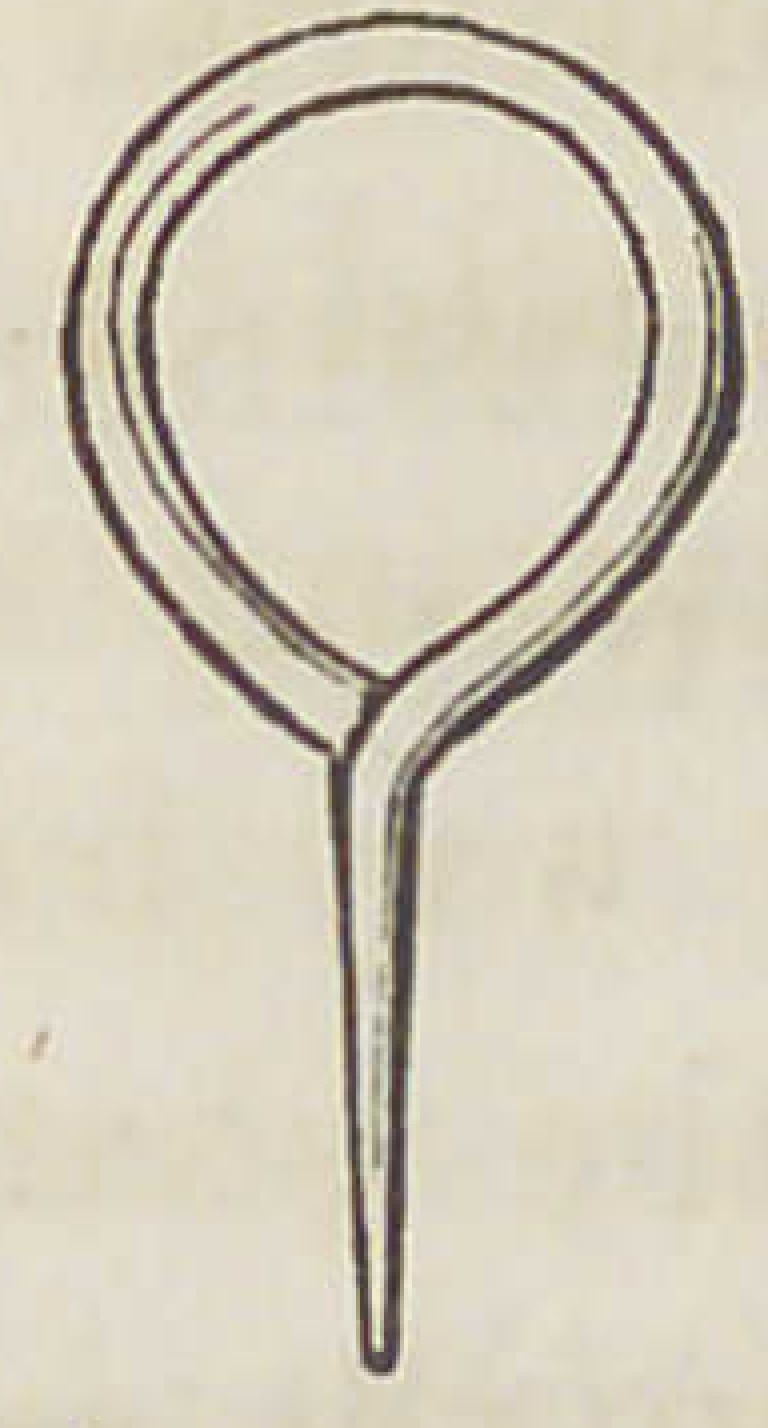


**Fig. 2. f2:**
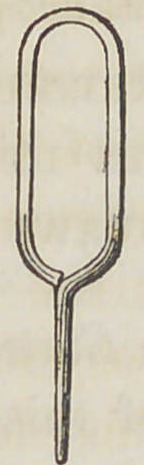


**Fig. 3. f3:**
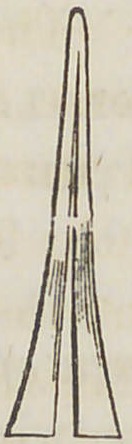


**Fig. 4. f4:**
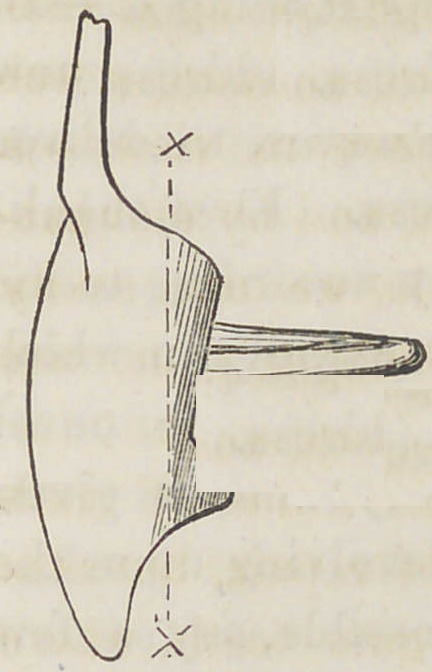


**Fig. 5. f5:**
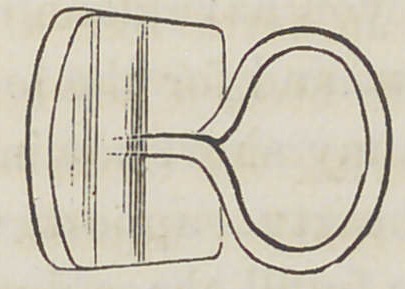


**Fig. 6. f6:**